# An investigation on the prevalence of microplastic in commercial and open pan salts obtained from Cox's Bazar and Maheshkhali region of Bay of Bengal (Bangladesh)

**DOI:** 10.1002/fsn3.3486

**Published:** 2023-06-15

**Authors:** Debapriya Mazumder, Md. Fahad Bin Quader, Suvanker Saha, Md. Ashraful Islam, Rakha Hari Sarker, Arpan Mitra Chowdhury

**Affiliations:** ^1^ Department of Applied Chemistry & Chemical Technology Chattogram Veterinary and Animal Sciences University Chattogram Bangladesh; ^2^ Department of Botany University of Dhaka Dhaka Bangladesh; ^3^ One Health Institute Chattogram veterinary and Animal Sciences University Chattogram Bangladesh

**Keywords:** Bangladesh, FTIR, microplastics, public health, Raman, salts, SEM, stereomicroscope

## Abstract

Unrestrained utilization of plastic has reached an intemperate state, menacing environment and human lives. The preliminary focus of this research was to investigate and divulge the contemporary status of microplastics (MPs) in commercialized and open pan salts from Cox's Bazar and Maheshkhali channels. A total of 27 samples were obtained. The samples were analyzed for the prevalence of MPs by FTIR and Raman spectroscopy (RS); the prevailing amount, color, size, and shapes were analyzed by stereomicroscope and SEM. The abundance of high‐density polyethylene, polyethylene terephthalate (PET), and low‐density polyethylene (LDPE) were detected by FTIR, meanwhile exuberance of cellulose acetate, polypropylene, PET, LDPE, and Nylon 6 were identified by RS. The average quantifications of MPs in Cox's Bazar, Maheshkhali, and packaged salts were found to be 6851.11 ± 538.18, 5638.89 ± 1001.18, and 3405.56 ± 638.57 per kg, respectively. ANOVA resulted in highly significant association between MPs and sampling sites (*p* = .001*). Post hoc Tukey's test revealed prominent link between commercialized and open pan salts based on the amount of MPs (*p* = .001*). The most prevalent colors were purple (28%) and blue (27%). The most frequent shapes were fibrous (79%) and fragmented (19.9%) MPs. The smallest MP was detected in commercial salt (1.55 μm), nearly identical and closer to the size of nanoplastics.

## Graphical abstract

Prevalence of microplastic is notably higher in open pan salts compared to packaged ones. Presence of microplastics in salts was confirmed by FTIR and Raman spectroscopy, while the microscopic images were confirmed with stereomicroscope and scanning electron microscope. One‐way ANOVA and post hoc Tukey's tests were performed to investigate the potential risk factors behind microplastic pollution in salts in Bangladesh.

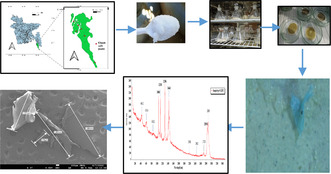

## 1 INTRODUCTION

An unprecedented elevation in the mass generation of plastic has occurred since 1950, which has surpassed 330 million tons presently (PlasticsEurope EJPE, 2016). The environmental problem posed by pollution of marine environment with plastics has been acknowledged by scientists on massive level and global extensive attention toward the issue has been devised (Barrett et al., 2020). Objects that are made of plastics such as packaging material and beverage bottles are examples of dynamic apparent debris and they are recognized globally for their notoriety as unsightly disgrace and potential threat to both nature and wildlife animals and humans. The term “white pollution” brought on by huge amount of goods designed for a single time use – which is convenient for customers, such as polythene bags – has garnered a lot of concern among researchers worldwide (Jiang et al., 2019), posing a major threat to the environment. These great quantities of remnant plastics have been observed to have negative impacts on plants, nature of the soil, and nutrients, hence the dynamic phrase has shifted from “white revolution” to “white pollution” (Liu et al., 2014).

The plastics which were not salvaged or irrecoverable (30.8%), after the combined recovery (69.2%), were thrown or dumped without any regard for safety (Ivleva et al., 2017). A significant amount of these trash materials invariably pollute the marine habitats and environment (Dris et al., 2015).

Microplastic (MP) is a term for plastic trash found in the environment that is usually visible on a microscopic scale, below 5 mm in size (Underwood et al., 2017). MPs have the capacity to act as hazardous or harmful pollutants due partly as a result of their microscopic length and organic nature (Tagg & do Sul, 2019).

Aquatic lives including marine organisms, tiny creatures, and juvenile stage organisms have the tendency to uptake these MPs in their growth phase, posing a significant risk to the marine ecosystem and aquatic environment (Conkle et al., 2018). One of the primary entry points for MPs from marine sources to human bodies is salt. It is also evident from many studies that processing phases of salt also contribute to the MP pollution in salt along with marine or aquatic source, beach sediments or sands, air or winds, etc. (Gündoğdu, 2018).

Marine plastic pollution occurs from either terrestrial sources (80% of maritime waste) or coastal practices (Coyle et al., 2020). Sources of MPs in the marine environment have been described in a recent study, where packaging materials derived MPs were found in the largest number (Ogunola & Palanisami, 2016).

The relevance and significance of the research arises with the fact and a strong possibility that these MPs may enter human bodies through the diet that includes daily intake of salt; a particular study conducted in the region of Bangladesh depicted the consumption of salt in adult population falls in the range of 15–21 g/day which is remarkably higher than the daily allowance of salt intake recommended by WHO and FAO (5 g/day) (Zaman et al., 2017). With the escalation in the consumption of salt in Bangladesh, the potential risk of being exposed to MPs increases significantly for people. The purpose of the research is to detect and quantify various types of MPs in salt collected from salt pans in Cox's Bazar and Maheshkhali region of Bay of Bengal (due to suitable geographical positioning and comparatively escalated yearly manufacturing rate) and commercial branded salts from Cox's Bazar metropolitan city and to disseminate provided that the result is alarming and restrict the unrestrained utilization and dumping of plastics at an early stage while in Bangladesh.

## 2 MATERIALS AND METHODS

### 2.1 Sample collection

**FIGURE 1:**
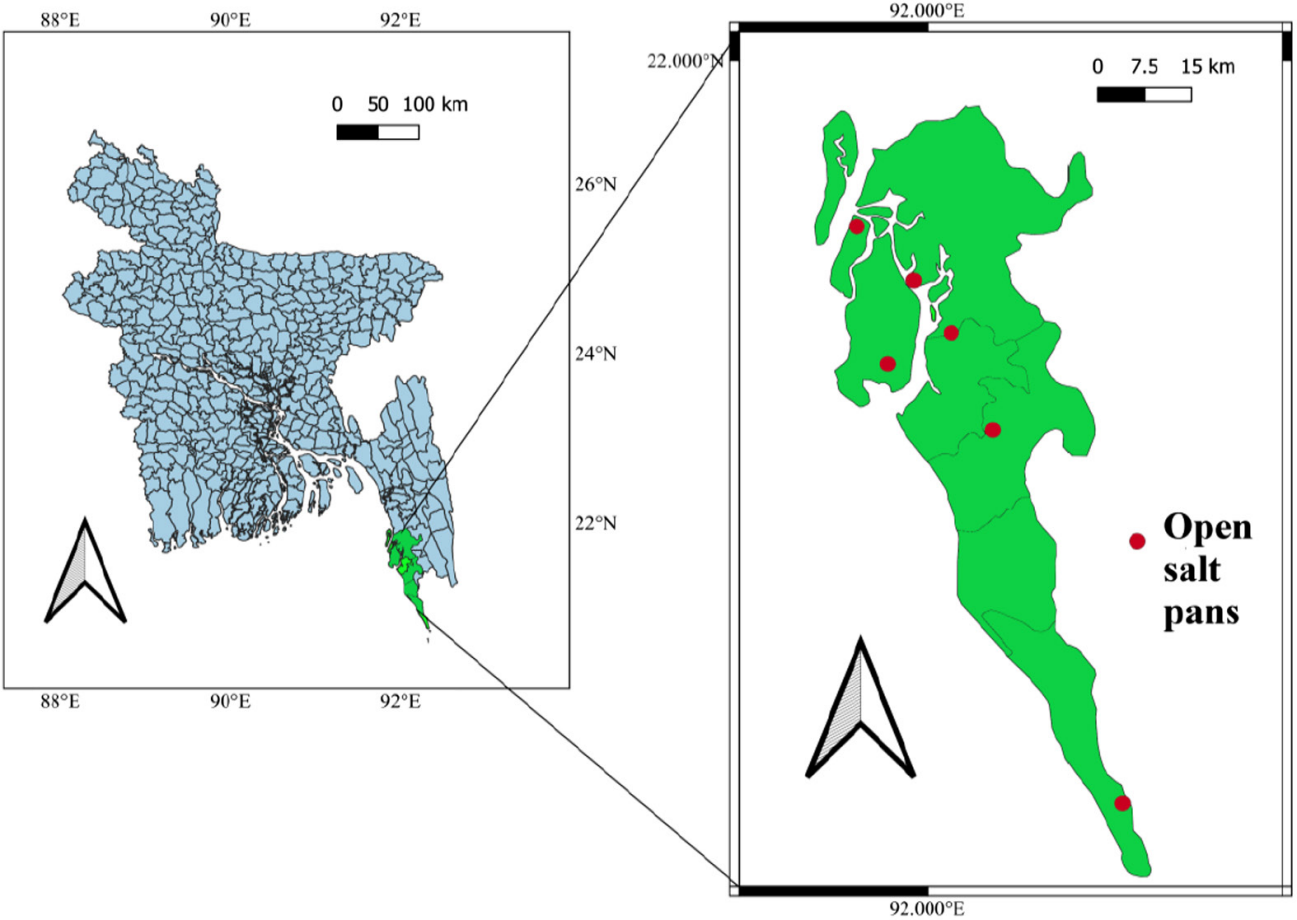
Geographical positioning of the sites for the collection of the specimens.

A total of 27 salt samples were gathered to propagate the experiment. There were a total of nine samples provided by Cox's Bazar region; locations including Teknaf, Ramu, and Napitakhali all contributed three samples each from separate pans to the tally. Furthermore, Maheshkhali region supplied a total of nine samples from Boro Maheshkhali, Matarbari, and Badarkhali; these open pan salts were collected by random sampling method. Samples of nine packaged brands (three sets of sample × 3) of refined salts were collected from the market in Cox's Bazar city by cluster sampling where each set included three samples with roughly the similar geographical positioning for raw salt processing (Figure 1).

### 2.2 Sample preparation and segregation of MP from salt

The samples were prepared with a little optimization and modification and followed the method described in similar studies (Parvin et al., 2022). In order to assimilate the organic matter, approximately 100 g of salt sample was blended with 100 mL of 30% H_2_O_2_ solution and kept in a shaker incubator at 65°C, 80 rpm for 24 h. Approximately 600 mL of deionized water (passed through cellulose nitrate filter paper to impede presence of any undesirable foreign particle including MP) was appended to the solution to dissolve the remaining salt samples. The solution was further transferred to a vacuum pump in order to filter the solution. Cellulose nitrate (CN) filter paper (Sartorius, Germany – 0.22 μm pore size, 47 mm diameter) was used because of its biobased option to remove particles (Ottenhall et al., 2018). After completion of the filtration process, the filter papers were dried at room temperature for an hour and stored in glass made Petri dishes inside an incubator with the temperature fixed at 20°C to circumvent any loss of dried off MPs until further analysis. The digestion resistant particles were found on the filter papers. In addition, a blank deionized water sample with no salt solution was passed through the filter paper and examined to eliminate the probable chances of operational contamination. There was no trace of MPs found in the blank samples of deionized water.

### 2.3 Stereomicroscopic observation of MPs

**FIGURE 2:**
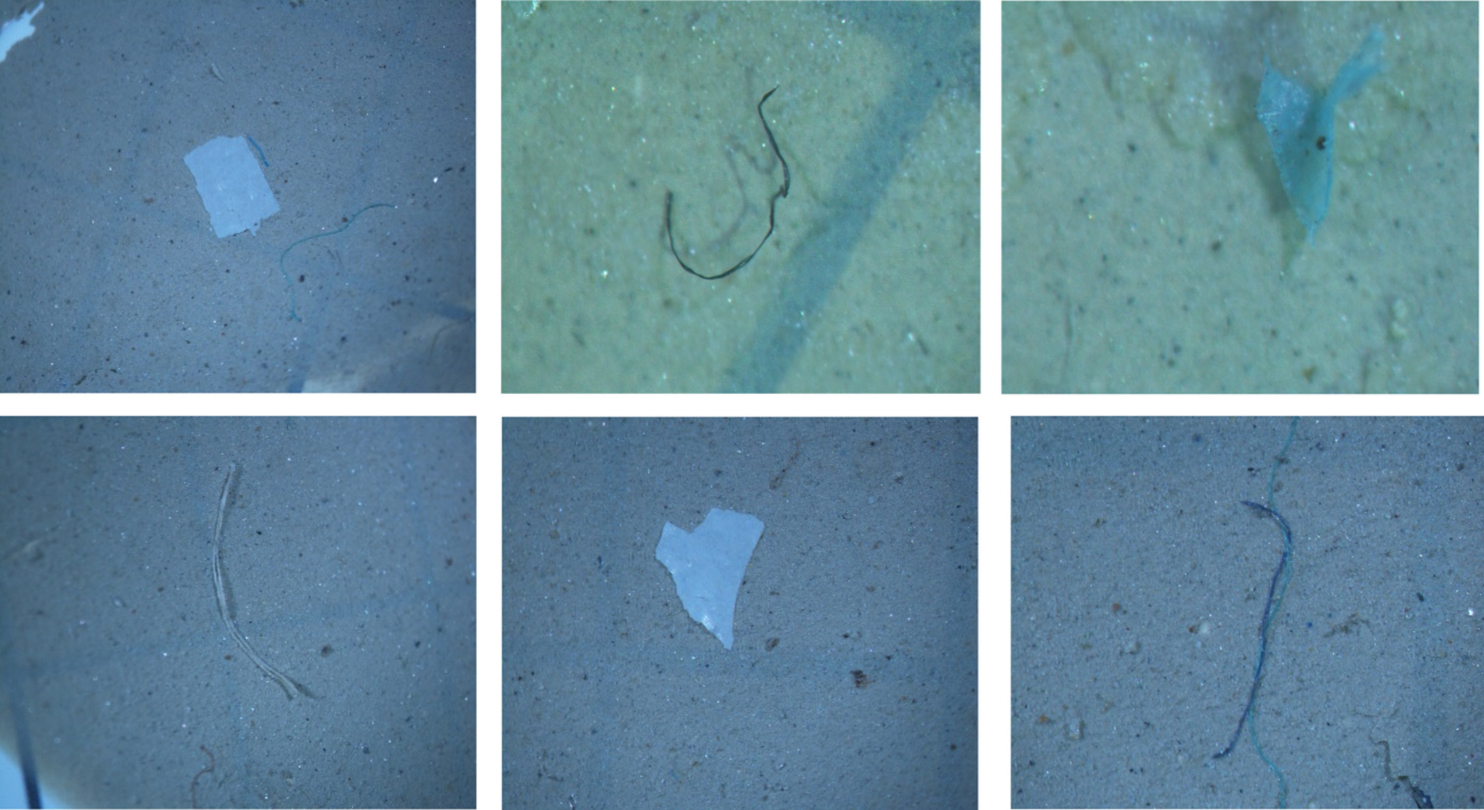
Stereomicroscopic images of MPs.

Cellulose nitrate filter papers were discerned utilizing a stereomicroscope (Leica® EZ4 HD) with a built in camera and magnification 8–35×. Images were captured to visualize and distinguish the MPs based on their morphotypes including size, color, and shape. MPs were further categorized into fiber, filaments, foams, and fragments (Karami et al., 2017). A total number of 18 visually “distinguishable” and “isolatable” MPs were segregated based on their distinguishable external appearance for the identification purpose (Figure 2).

### 2.4 High‐resolution imaging and particle size analysis by scanning electron microscope (SEM)

**FIGURE 3:**
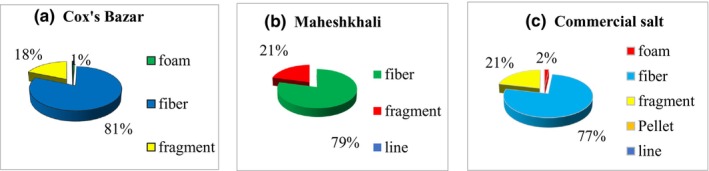
Scanning electron microscopic images with size of MPs.

The patterning of the surface of the MPs and photographs along with size measurement were arbitrated by a high‐resolution SEM (Eriksen et al., 2013). JSM‐7610F field emission SEM was utilized for the completion of the purpose. A magnification range of 50–3000× was pertained for imaging and particle size measurement (Figure 3).

### 2.5 Fourier‐transformed infrared spectroscopy–attenuated total reflectance (FTIR‐ATR) analysis

FTIR (PerkinElmer, IR spectrum ES version 10.6.2) was used to perform the identification of MPs. ATR method was applied to measure the samples. FTIR‐ATR ascertains the presence or absence of functional groups and structures of the polymers (Anderson & Voskerician, 2010). The wave number was set from 4000 to 400 cm^−1^ for the spectral range and the resolution was set at 4 cm^−1^ for the spectral resolution with a slight modification of a similar study (Hossain et al., 2019). The spectra were later assigned to compare with automated library in the FTIR (Parvin et al., 2022). To circumvent the occurrence of false negative results, the spectra were also compared to the absorption bands of each plastic polymer in existing literatures.

### 2.6 Identification of MPs by Raman spectroscopy

MPs that were diminutive for FTIR analysis or the particles that could not be desiccated properly were subjected to Raman spectroscopy (RS; Li et al., 2018). RS allows for the analysis of MP particles that have been visually or microscopically sorted. A Horiba MacroRam with excitation laser 785 nm, laser power <5 mW was applied for the identification purpose. A total of seven sample types segregated from the filter paper were analyzed by RS.

### 2.7 Procedural contamination control

To evade the procedural contamination, precaution in every step was taken into account. To avoid plastic contamination, plastic bags were not used in the process. Petri dishes were covered after transferring the filter papers to circumvent air‐borne contamination referred by another study (Devriese et al., 2015). Both aerial contaminants and synthetic fibers can cause cross contamination in the process.

### 2.8 Statistical analysis

Statistical analysis was accomplished on STATA 13® software and analysis of variance (ANOVA) and post hoc Tukey's test were performed.

## 3 RESULTS AND DISCUSSION

### 3.1 MP based on shapes

**FIGURE 4:**
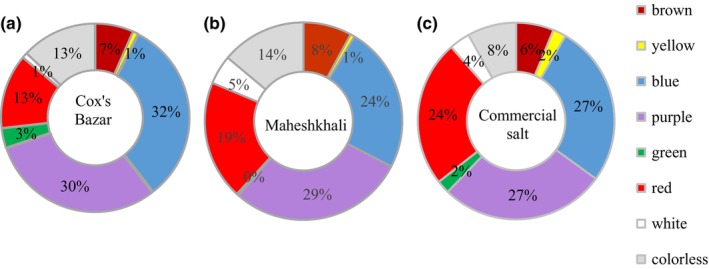
Different shapes of MPs in open pan and commercial salts [a, b, c (from left to right) depicting the salt samples obtained from Cox's Bazar, Maheshkhali, and Commercial markets, respectively]. (a) Different shapes of MPs in salt samples collected from open pans in Cox's Bazar, Bangladesh; (b) different shapes of MPs in salt samples collected from open pans in Maheshkhali; (c) different shapes of MPs in commercial salt samples collected from markets.

There was a substantial number of fiber‐shaped MP (5502.2 ± 463.32 particles/kg) detected in the salt samples from Cox's Bazar (81%). Fragmented MPs comprised the second position with the amount of 1254.4 ± 242.08 particles/kg (18%) (Figure 4a).

Microfibers were the most abundant MP (4455.56 ± 854.93 particles/kg) spotted in the samples from Maheshkhali (79%), followed by fragment‐shaped MP accounting for 1183.33 ± 298.96 particles/kg (21%) (Figure 4b).

Approximately 77% of the MPs observed in the commercial samples were microfiber in morphological nature (2622.22 ± 682.42 particles/kg) (Figure 4c).

### 3.2 MP based on colors

**FIGURE 5:**
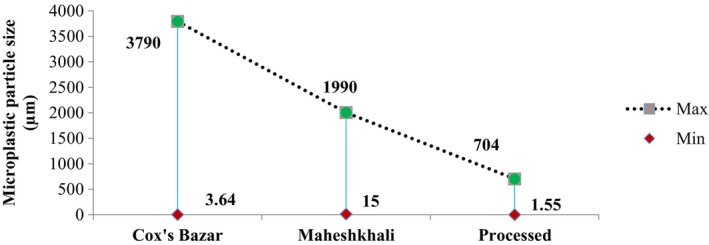
Different colors of MPs in open pan and commercial salts.

In the context of colored MP, blue and purple hues were found to be the most prevalent in the samples from Cox's Bazar (2188.89 ± 657.43 particles/kg salt were blue [32%] and 2072.22 ± 514.85 particles/kg were purple [30.2%]. The other two major colored MPs observed were red (883.33 ± 345.51/kg [13%]) and brown (451.11 ± 142.34/kg [7%]) (Figure 5).

Approximately equivalent amounts of purple (1638.89 ± 613.79 particles/kg), blue (1361.11 ± 294.51 particles/kg), and red (1105.56 ± 320.59 particles/kg) and colorless (788.89 ± 219.06 particles/kg) were identified in the salt samples gathered from Maheshkhali representing 29%, 24%, 19%, and 14%, respectively.

The MPs in the commercial samples included an equal dispersion of both blue (855.56 ± 251.80 particles/kg) and purple (877.78 ± 206.32 particles/kg), each representing 27% of the total samples. A considerable proportion of red MPs (24%) were observed to be present in the samples (783.33 ± 280.62 MPs/kg).

### 3.3 MP particle size

**FIGURE 6:**
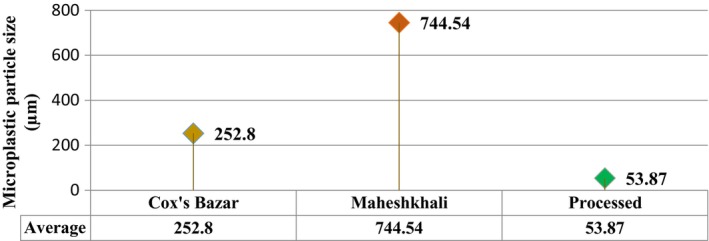
Maximum and minimum particle size.

**FIGURE 7:**
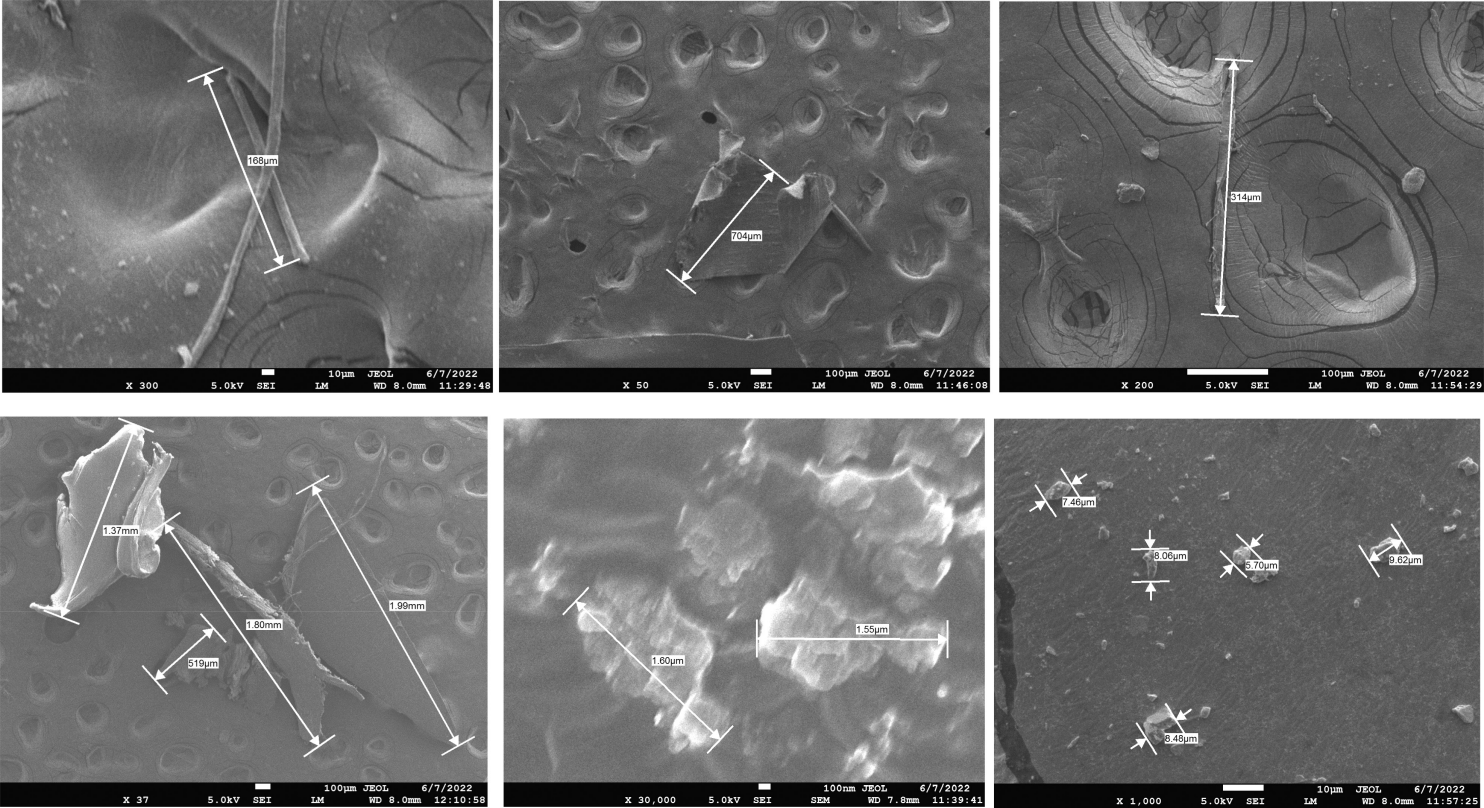
Average size of MPs.

SEM was utilized to ascertain the MPs' particle sizes. Sizes of the MP particles have been analyzed based on three parameters including maximum, minimum, and average size (Figures 6 and 7). The sizes ranged from 3.64 to 3790, 15 to 1990, and 1.55 to 704 μm in the salt samples acquired from Cox's Bazar, Maheshkhali, and commercial brands, respectively. Average size distributions of the MPs in the samples from Cox's Bazar, Maheshkhali, and commercialized salts were 252.8, 744.4, and 53.87 μm, respectively.

### 3.4 Quantification of MPs

**FIGURE 8:**
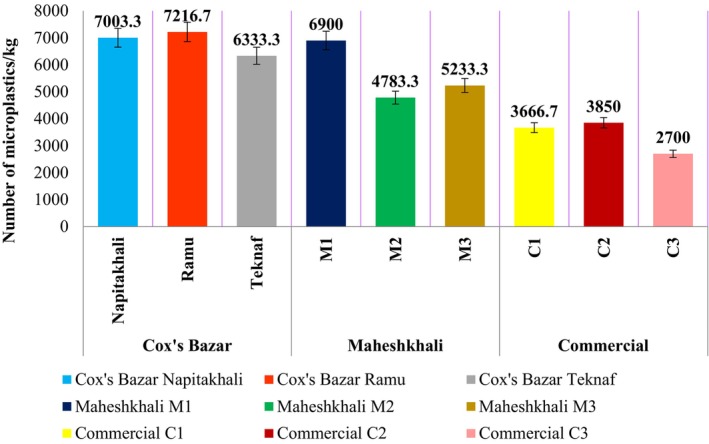
Quantification of MPs.

The total number of MPs ranged from 2700 to 7216.7 particles/kg in different salt samples, which gave an average of 5298.52 ± 1624.163 MPs/kg collectively for salt acquired from the salt pans and commercialized salts (Figure 8, Table 1).

**TABLE 1:** Abundance of MPs.

Area	Location	Amount of MPs (mean ± standard deviation)	Mean ± SD	Frequency
Cox's Bazar	Napitakhali	7003.33 ± 521.57	6851.11 ± 538.18	3
	Ramu	7216.67 ± 160.73		3
	Teknaf	6333.33 ± 472.58		3
Maheshkhali	Boro Maheshkhali	6900 ± 200	5638.89 ± 1001.18	3
	Matarbari	4783.33 ± 381.88		3
	Badarkhali	5233.33 ± 305.51		3
Commercial	C1	3666.67 ± 401.04	3405.56 ± 638.57	3
	C2	3850 ± 312.25		3
	C3	2700 ± 476.97		3

The average results for MPs quantified in the salts from open pans were recorded as 6851.1 ± 538.18 MPs/kg in Cox's Bazar and 5638.89 ± 1001.18 MPs/kg in Maheshkhali region.

The count of MPs recorded in the commercialized salt contained the lowest of all the other samples (2700–3670 particles/kg), resulting in an average of 3405.6 ± 638.57 MPs/kg.

### 3.5 Identification of the MPs

**FIGURE 9:**
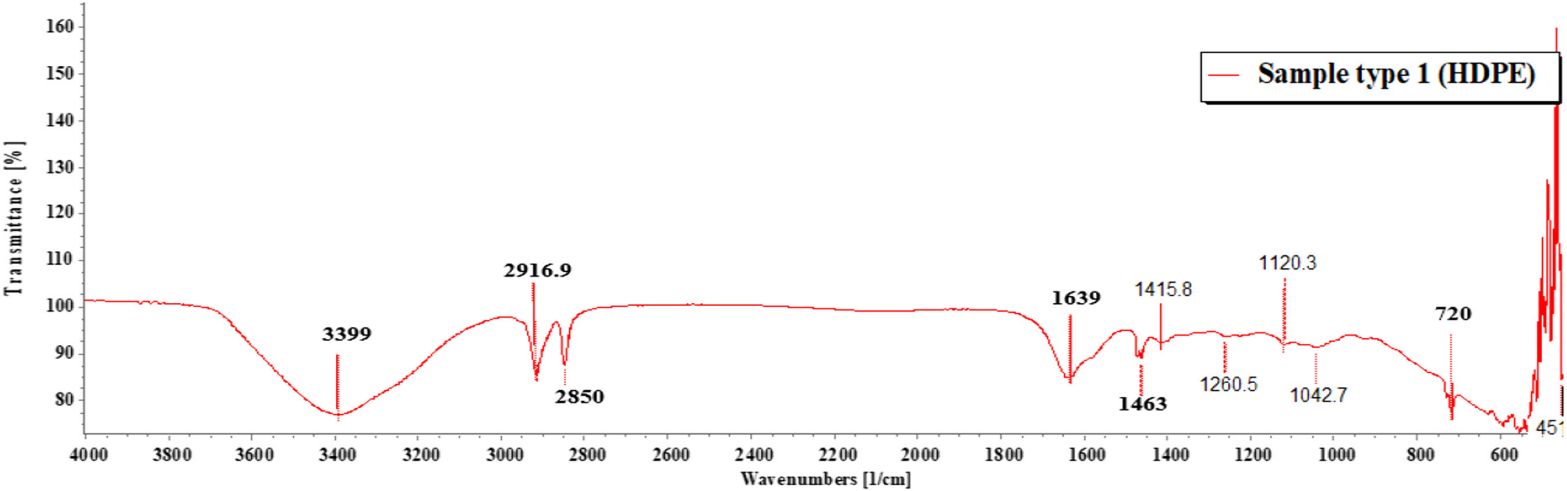
Sample type 1 (HDPE).

**FIGURE 10:**
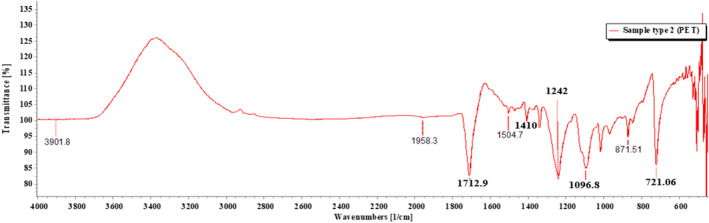
Sample type 2 (PET).

**FIGURE 11:**
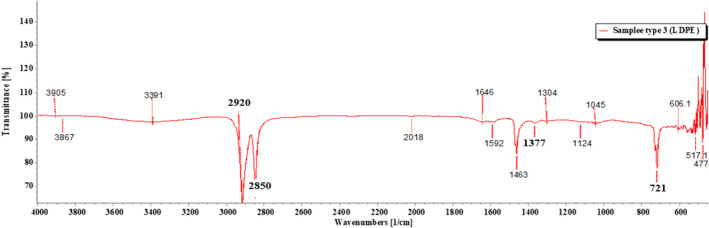
Sample type 3 (LDPE).

**FIGURE 12:**
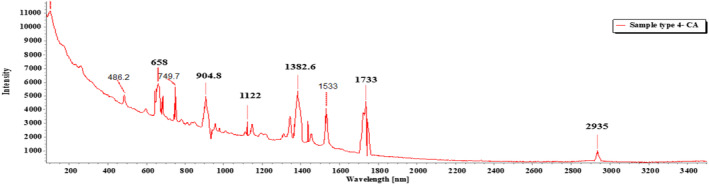
Sample type 4 (CA).

**FIGURE 13:**
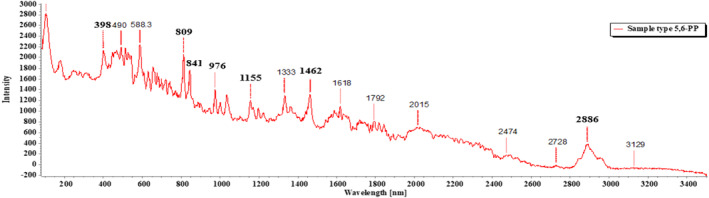
Sample types 5 and 6 (PP).

**FIGURE 14:**
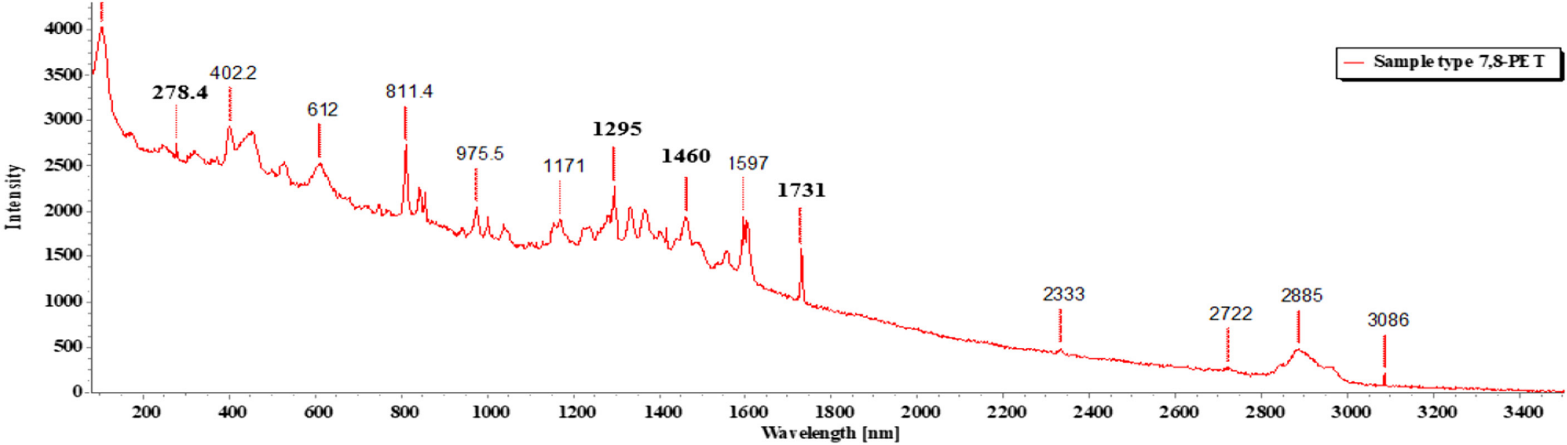
Sample types 7 and 8 (PET).

**FIGURE 15:**
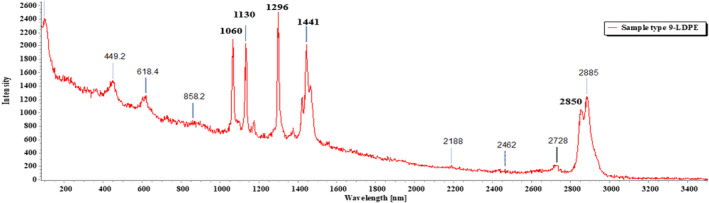
Sample type 9 (LDPE).

**FIGURE 16:**
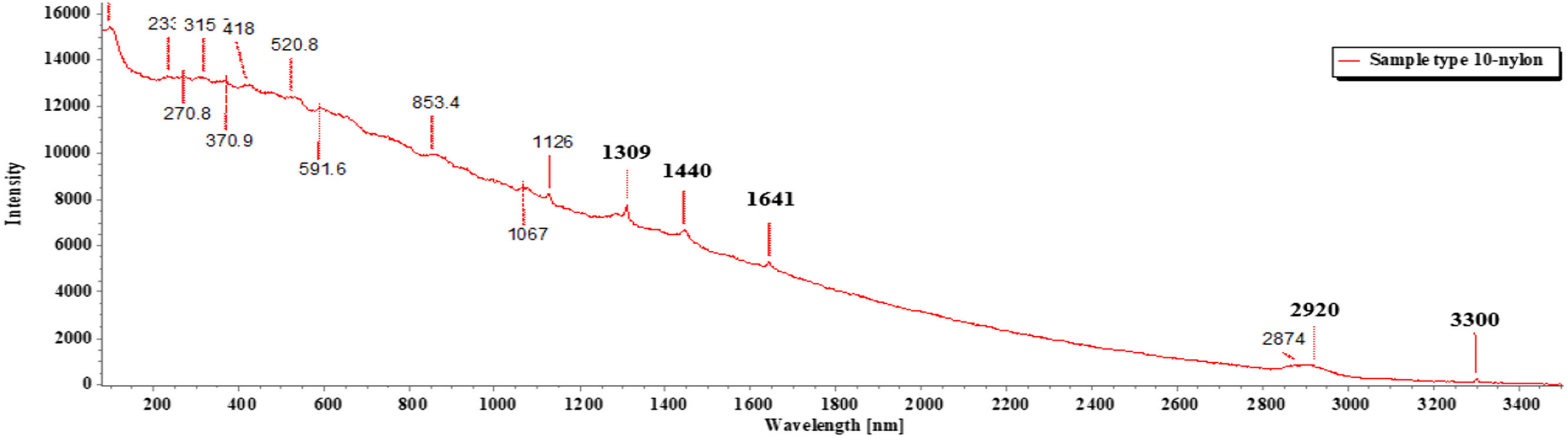
Sample type 10 (Nylon 6).

Of the 18 segregated MP particles, all MPs were identified and fallen into 10 categories and numbered from 1 to 10, based on their similar external appearance. The rate of success for the identification of MP particles was 100%. In the FTIR examination, only three types of polymers (1–3) were recognized. RS was performed on the left out samples (4–10) that remained unidentified in the FTIR. MP sample types 1–3 were identified with FTIR‐ATR. The identification resulted in the prevalence of high‐density polyethylene (HDPE), polyethylene terephthalate (PET), and low‐density polyethylene (LDPE), respectively, for MP sample types 1, 2, and 3 (Figures 9, 10, 11; Greene, 2021; Jung et al., 2018; Noda et al., 2007; Sahu et al., 2019). MP sample types 4–10 were subjected to RS for their distinctive size differences. The identification of the aforementioned samples resulted in the existence of cellulose acetate (CA), polypropylene (PP), PET, LDPE, and Nylon 6, respectively (Figures 12, 13, 14, 15, 16; Andreassen, 1999; Bailey et al., 1977; Boerio et al., 1976; Cho, 2007; Gall et al., 1972; Ibrahim & He, 2017; Kida et al., 2016; Martínez‐Romo et al., 2015; Minogianni et al., 2005; Sánchez‐Márquez et al., 2015; Stuart, 1996; Xu et al., 2019). Both the identifications were carried out by FTIR and Raman libraries and comparing the significant peaks with existing literatures of the standard plastic polymers.

### 3.6 Statistical analysis

Statistical analysis was accomplished on STATA 13® software. Descriptive analysis was carried out with histogram plotting. Upon finding the normal distribution of the samples, ANOVA was performed (Table 2).

**TABLE 2:** ANOVA based on total amount of microplastics.

Area	Mean	SD	*p* (between groups)
Cox's Bazar	6851.11	538.18	.001*
Maheshkhali	5638.89	1001.18	
Commercial salt	3405.56	638.57	

* Highly significant.

ANOVA substantiated statistically significant corelation between locations or sampling sites with MPs load in the salt samples. Post hoc Tukey's test with 95% confidence interval was implemented to investigate the relation between the MP burdens of salt samples and geographic locations or intercomparison among each location themselves. Pairwise comparison was established with Tukey's test (Table 3).

**TABLE 3:** Post hoc Tukey's test of pairwise comparison.

Total microplastics	Tukey		Tukey's 95% CI	
	Standard error	*p*		
Maheshkhali vs. Cox's Bazar	354.8369	.006*	−2098.35	−326.93
Commercial salt vs. Cox's Bazar	354.8369	.001*	−4331.69	−2559.42
Maheshkhali vs. Commercial salt	354.8369	.001*	−3119.46	−1347.20

* Highly significant.

## 4 DISCUSSION

### 4.1 Factors affecting abundance of MPs in different sampling zones

The amount of MPs in salt in this study, collectively in both open pan and commercial salts, is higher (5298.52 ± 1624.163 MPs/kg) than previously carried out study which resulted in 2676 MPs/kg (Parvin et al., 2022). A similar study was carried out in the same region with slightly different outcome and significantly lower MPs than the result of this study, 28.53 ± 2.43 to 93.53 ± 4.21 MPs/kg (Islam et al., 2022). Compared to studies carried out globally, the amount of MPs in this study was lower (23–115 MPs/200 g in Gujrat and Tamil Nadu (Vidyasakar et al., 2021), 13.2 ± 6 MPs/50 g in China (Li et al., 2020), 50–280 MPs/kg in Spanish salt (Iñiguez et al., 2017)). The potential reason behind the escalated amount of MPs in salt may arise from the fact that unrestrained dumping of plastic is common in Bangladesh especially in tourism state like Cox's Bazar (Pushan et al., 2022). Another potential reason behind the soaring amount of MPs may be the establishment of coal‐based Matarbari power plants and deep sea port in the Maheshkhali region.

The ANOVA of the samples (*N* = 27) resulted in a significant *p* (.001*) which was evidential to the existence of notable association between the sampling sites and amount of MPs present in salt samples. The salt samples gathered from Cox's Bazar contained more MPs/kg than the samples acquired from Maheshkhali. Prior studies revealed a significant association between MP pollution and tourism (Garcés Ordóñez et al., 2020; Wei et al., 2022). Furthermore, tourism was identified as one of the reasons for MP pollution in the Cox's Bazar region (Pushan et al., 2022). According to data available, during the peak travel season, Cox's Bazar attracts approximately 2 million visitors and pollution index of MPs is high (Dey et al., 2015; Mahmood et al., 2022; Pushan et al., 2022). Comparatively, Maheshkhali region contained lower amount of MPs (Tukey's test, *p* = .006). This might result from the fact that the number of tourists is markedly smaller in Maheshkhali region (Mahmood et al., 2022). Maheshkhali also faces modest level of disruption caused by humans. It is universally acknowledged that fishing practices are significant contributor to the MP pollution (Jeyasanta et al., 2020). There are more research with findings that are analogous to these of this study (Jayasiri et al., 2013; Rahman et al., 2020). Commercialized salts were detected containing comparably lesser MPs per kilogram (*p* = .001 in Tukey's test when compared to Cox's Bazar and Maheshkhali region) because refined salts go through a line of industrial process and are filtered (Lanka et al., 2014).

### 4.2 Different morphological properties of the MPs

Recognition of the plastic origins can be facilitated by the differences in the colors of the MPs (Zhang et al., 2020). The varied hues of MPs detected in all the collected salt samples are ranked in the descending order: purple > blue > red > colorless > brown > white > green > yellow. There have been relevant studies revealing an abundance of white and transparent MPs in the most frequent amount (Martí et al., 2020). Blue MPs resulted from the fishing net frequently used in the marine environment and the finding of this study was observed to be identical to the findings of the study conducted in Thailand (Kasamesiri et al., 2021). Decolorization and pale color of the MPs can be caused by UV radiation and hydraulic impacts; these might be the reasons for the existence of such pale colored MPs, such as white (Ren et al., 2020). The rest of the colors of MPs are sourced from plastic materials widely used in the day to day lives. Following the feeding of birds, vacationers and travelers were spotted tossing nonbiodegrading packages and trashes into marine water (Bhuyan et al., 2019), which eventually contaminated the salt.

The fibrous MPs were determined to be the most prevalent one, followed by fragmented MPs. Fibrous MPs are commonly produced by polyamide or nylon‐based strings, even clothing materials (Kasamesiri et al., 2021). There are some potential sources of fibers including mesh utilized in the fishing purpose, gearing appliances, and fabric‐based goods that get mixed with sewage water, outflow of river or lake waters, inundation, and consequently contaminate salt pans (Hossain et al., 2021). The result demonstrated a close connection to the previously conducted research (Kasamesiri & Thaimuangphol, 2020; Martínez Silva & Nanny, 2020; Yuan et al., 2019). The macroplastics dumped by humans degrade in the environment leading to the formation of fragmented MPs (Widiastuti et al., 2021). External and interior air in general can include fragment‐shaped and fibrous MPs derived from clothing materials which are biologically assiduous. Substantial evidences have been found that MPs, measuring up to 1650 μm, are prevalent in the outside air (Gasperi et al., 2018).

The result of a considerable number of studies was consistent in salt samples regarding size of MPs at upper end of the scale (Renzi & Blašković, 2018), including studies carried out in Bangladesh (Pushan et al., 2022; Rakib et al., 2021). The marketed salt samples contained significantly smaller MPs (1.55–704 μm) than those detected in the salt from the open salt pans. The findings were pertinent to recent studies conducted in Bangladesh (Parvin et al., 2022; Zafar et al., 2020). Salt manufacturing includes an integral step called grinding to ensure homogeneity in size (Iñiguez et al., 2017). It might be possible for the MPs to have shrunk significantly in the commercial salt samples by the mechanical forces induced by grinding. The clearly visible MPs of 1–5 mm in length detected in the study were potential examples of hastened disintegration by wave or UV rays (Hossain et al., 2021).

### 4.3 Probable sources of the identified MPs

A research was carried out throughout Cox's Bazar and the consequence was congruent to the findings of this study, the researchers discovered HDPE, polystyrene (PS), PET, EVA, nylon in salt (Parvin et al., 2022). A significant study in the Maheshkhali coastal area investigated MP accretion in salt, which led to the discovery of PE, PET, PS, and PP (Rakib et al., 2021). Cox's Bazar zone was the focus of another MPs assessment, which concluded that shore soils and ocean waters from surface possessed an overabundance of approximately 42,800,000 particles and 21,700 particles/km^2^, respectively (Mahjabin et al., 2020). This is an unequivocal and resounding indication that high concentration of MPs from shore sands and ocean water can interchangeably escalate into the salt manufactured in the nearby region (Parvin et al., 2022).

The MPs identified through RS was found synonymous yet exclusive to the findings of preexisting studies (Khuyen, Dinh‐Vu, et al., 2021; Khuyen, Le, et al., 2021). According to past finding, the MPs that are frequently discovered in salt are PE and PET (Danopoulos et al., 2020).

HDPE and LDPE could have originated from carrier bags or similar packaging materials; PET could have arisen from clothing or fragmented bottles or similar sources. The plastic made caps that were placed on bottles might have been one possible contender for PP source. MPs from cigarette filters might release as CA straight into environment, nylon might have derived from fishing nets (Deepthi et al., 2018; Gerritse et al., 2020; Joly & Coulis, 2018; Shah et al., 2008). Another potential source of MPs may be the raw packaging and clothing deriving from the workers in the Matarbari coal‐based power plants and deep sea port. It may be considered as one of the factors that completion of the humongous projects as these require many packaging, clothing, and synthetic plastic materials which further pollute the environment and escalate the amount of MPs in the salt production areas of the region. Amid the current abysmal of Bangladesh, it is difficult to halt the production or use of plastic; but it can be suggested that limiting the use and dumping of plastic made materials, campaigning against MP pollution, and concrete laws to minimize the pollution can be implemented. Moreover, it is mandatory that people in general are introduced more to the biodegradable and recyclable plastics (Deepthi et al., 2018).

## 5 CONCLUSION

The sole purpose of this study was to determine how perilous and profound the prevalence of MPs was in salt since daily use of salt is ubiquitous among people. The experiment on salt sample was rational due to the fact that people consume salt without subjecting it to any sort of pretreatment. The outcome of the study revealed a potentially catastrophic repercussion. Both of open pan and commercialized salts contained MP burden ranging from 2700 to 7216.1 MPs/kg. It is reasonable to deduce based on the findings of this study that the likelihood of humans being exposed to MPs on regular basis through the route of salt ingestion rises to and between 13.5 and 36 particles contingent on the quantity of salt consumed. The smallest MP retrieved was 1.55 μm in length, remarkably synonymous and closer in size to nanoplastics. The study incomparably furnishes to the portrayal of the existing MP pollution in salt and possible tracts and channels of contamination. In addition, the findings reported here might be valuable and informative to the environmentalists and policymakers to help the associated authorities in lessening the MP burden on salts to a certain extent. The authorities concerned are suggested to make and implement policies on limiting the use of plastic materials and unplanned dumping of these materials. From the perspective of food safety, the people are encouraged to shift to the commercial salts because these were found slightly safer in comparison to the open pan ones. The manufacturing companies are urged to execute filtering with the lowest pore size filter papers, preferably pore size below 0.1 μm, as a preliminary process. Furthermore, it is suggestion to construct or relocate the salt manufacturing sites away from tourist spots. Campaigning against plastic pollution is also suggested to minimize the plastic usage.

## AUTHOR CONTRIBUTIONS

**Debapriya Mazumder:** Conceptualization (lead); data curation (lead); formal analysis (lead); funding acquisition (equal); investigation (lead); methodology (lead); project administration (equal); resources (lead); software (lead); supervision (equal); validation (lead); visualization (lead); writing – original draft (lead); writing – review and editing (lead). **Md. Fahad Bin Quader:** Conceptualization (supporting); funding acquisition (supporting); methodology (supporting); project administration (supporting); resources (supporting); supervision (supporting); writing – review and editing (supporting). **Suvanker Saha:** Conceptualization (equal); data curation (supporting); funding acquisition (supporting); methodology (equal); project administration (supporting); supervision (supporting); validation (supporting); writing – review and editing (supporting). **Md. Ashraful Islam:** Conceptualization (supporting); funding acquisition (supporting); project administration (supporting); supervision (supporting); writing – review and editing (supporting). **Rakha Hari Sarker:** Conceptualization (supporting); investigation (supporting); project administration (supporting); software (supporting); supervision (supporting); writing – review and editing (supporting). **Arpan Mitra Chowdhury:** Conceptualization (supporting); methodology (supporting); resources (supporting); visualization (supporting).

## FUNDING INFORMATION

The research was funded by fellowship granted to the corresponding author Debapriya Mazumder by the Ministry of Science and Technology, Bangladesh (NST 2019–20, Merit: 488, ID: 2663).

## CONFLICT OF INTEREST STATEMENT

There is no conflict of interests among the authors associated with this study.

## ETHICS STATEMENT

The study did not require to obtain any ethical approval.

## PERMISSION TO REPRODUCE MATERIALS FROM OTHER SOURCES

This study does not contain any materials reproduced from other sources.

## Data Availability

The data that support the findings of the study are available on request from the corresponding author. The data are not publicly shared due to privacy and ethical restrictions.
